# Supplementary material for: A Likelihood Approach for Real-Time Calibration of Stochastic Compartmental Epidemic Models
Source: PLoS Comput Biol. 2017 Jan 17;13(1):e1005257. doi: 10.1371/journal.pcbi.1005257 (PMC5240920; doi:10.1371/journal.pcbi.1005257)
Supplement: S1 File — (TAR.GZ) [file pcbi.1005257.s014.tar.gz › HSPH_Online-SI-Revision/Additions/EnKF08/n100-extreme-SEITR/n100-extreme-SEITR_EnKF_table0.pdf]

1	R0	{1.38654, 9.85565}	{2.63523, 9.65694}	{{2.15169, 3.9091}, {3., 13.0941}}	{90.0578, 38.0167}	inf	5261.	3310.81	{1982.35, 4623.83}	82.3843	0.788791	newly	12085.	2539.66	{1111., 5580.}	79.9424	0.76523	Reff	0.312221	0.619867	{0.291053, 1.14477}	105.577	ar	92379.	180481.	{168830., 191998.}	95.3701
2	R0	{2.36629, 12.2029}	{2.5409, 7.105}	{{2.26066, 2.39537}, {3., 10.679}}	{23.151, 41.7761}	inf	4672.	9293.99	{5256.58, 26716.7}	74.281	0.660418	newly	17453.	12064.	{3328., 49198.}	75.826	0.465082	Reff	0.202507	0.891272	{0.482288, 2.26064}	343.783	ar	99022.	211819.	{186460., 332148.}	113.912
3	R0	{2.32253, 12.836}	{2.12464, 5.1499}	{{1.58749, 3.92069}, {3., 6.48726}}	{34.773, 59.8792}	inf	6387.	1436.56	{159.243, 2309.32}	96.0423	1.57799	newly	18266.	1371.87	{118., 1929.}	92.4895	1.33117	Reff	0.29092	0.312286	{0.150886, 0.430662}	49.275	ar	98997.	176683.	{164580., 197219.}	78.4729
4	R0	{1.79075, 11.2058}	{2.61225, 7.32147}	{{2.11006, 2.41826}, {3., 11.8979}}	{45.8747, 39.4972}	inf	6785.	5488.31	{4674.31, 6190.7}	80.3905	0.715895	newly	16022.	5615.7	{2509., 7572.}	72.9177	0.57985	Reff	0.341174	0.479371	{0.128983, 0.753275}	74.0843	ar	96920.	188906.	{179181., 196234.}	94.9095
5	R0	{2.26952, 9.62371}	{2.37427, 4.97453}	{{1.58294, 3.77514}, {3., 6.42199}}	{29.2218, 48.3096}	inf	2810.	2719.81	{252.734, 2444.31}	91.2063	1.37211	newly	16920.	3190.66	{190., 2422.}	91.9987	1.19208	Reff	0.133902	0.348176	{0.02842, 0.430437}	182.048	ar	98881.	182640.	{166418., 237236.}	87.8665
6	R0	{1.57191, 6.89004}	{2.06783, 6.08186}	{{0.690417, 2.0665}, {3., 8.25209}}	{72.3708, 33.0449}	inf	4582.	2137.62	{59.6189, 3067.04}	94.8624	1.5473	newly	17405.	1856.07	{43., 4022.}	93.3343	1.54716	Reff	0.240125	0.349925	{0.0571756, 0.442851}	94.0423	ar	95046.	169226.	{159261., 181264.}	78.0466
7	R0	{1.99226, 17.2339}	{2.72027, 9.91566}	{{2.17728, 3.83457}, {3., 13.5656}}	{36.5421, 42.4642}	inf	5638.	2255.88	{1113.45, 2946.54}	92.4862	1.19444	newly	11332.	2230.72	{571., 2603.}	85.5411	0.951777	Reff	0.270071	0.679991	{0.424691, 0.941411}	154.496	ar	97992.	171915.	{163415., 180259.}	75.4379
8	R0	{2.63369, 15.1879}	{1.88849, 6.54568}	{{0.0976208, 2.07167}, {3., 10.1452}}	{43.0381, 56.902}	inf	3741.	7097.22	{3303.9, 8535.6}	82.1535	0.865118	newly	14766.	7077.96	{1927., 10441.}	80.7799	0.597448	Reff	0.159786	0.433393	{0.0179176, 0.854876}	205.577	ar	99451.	187956.	{173593., 193967.}	88.9937
9	R0	{2.53271, 9.90552}	{2.41656, 4.97999}	{{1.58023, 3.80803}, {3., 6.57912}}	{24.4501, 49.7251}	inf	2374.	3092.04	{937.017, 3561.18}	91.6407	1.26431	newly	17411.	3179.13	{667., 3725.}	89.8813	1.06656	Reff	0.11382	0.321923	{0.00963779, 0.544415}	214.079	ar	99355.	197170.	{181392., 249404.}	99.4272
10	R0	{2.48791, 18.9003}	{2.4427, 8.56151}	{{1.50358, 3.63257}, {3., 19.8736}}	{23.4483, 56.8032}	inf	6612.	10929.6	{955.219, 19388.4}	88.1304	1.05195	newly	13614.	10432.8	{655., 41391.}	110.859	0.816781	Reff	0.296509	0.642814	{0.0793423, 1.49973}	144.715	ar	99291.	198985.	{167646., 295748.}	100.406
11	R0	{2.84414, 18.4765}	{2.26541, 6.9302}	{{0.376711, 1.51292}, {3., 19.3861}}	{27.2528, 65.304}	inf	5335.	7686.53	{872.043, 7315.36}	86.6449	1.09753	newly	14675.	8684.98	{883., 17385.}	101.419	0.779064	Reff	0.221814	0.398519	{0.079098, 0.573646}	114.907	ar	99654.	195181.	{175408., 245357.}	95.8588
12	R0	{2.29369, 14.622}	{2.50365, 12.7351}	{{1.54111, 3.97937}, {3., 19.7684}}	{28.7335, 43.5388}	inf	4308.	11723.7	{1483.36, 20760.6}	86.2864	0.849393	newly	14159.	6374.2	{1018., 15644.}	86.8252	0.74716	Reff	0.188656	0.689011	{0.374764, 0.922313}	268.968	ar	98982.	206367.	{180587., 280403.}	108.489
13	R0	{2.73413, 16.7529}	{2.52377, 9.08872}	{{2.03807, 4.0999}, {3., 13.8878}}	{16.2511, 45.7484}	inf	4068.	7692.34	{1460.27, 11143.4}	87.224	0.961721	newly	14704.	8347.53	{1051., 13971.}	97.0327	0.722154	Reff	0.173699	0.832515	{0.438175, 2.03804}	380.716	ar	99543.	209503.	{184169., 292071.}	110.464
14	R0	{1.79554, 16.5943}	{2.49018, 11.1424}	{{1.61372, 3.52972}, {3., 17.1953}}	{45.8349, 35.4327}	inf	7555.	4458.43	{1258.75, 11674.8}	84.2118	0.95567	newly	11642.	4387.76	{636., 13574.}	76.5642	0.767819	Reff	0.390135	0.92455	{0.381163, 1.62877}	138.993	ar	96821.	199875.	{177405., 252115.}	106.438
15	R0	{1.91429, 13.7771}	{2.67842, 9.25689}	{{2.17545, 4.0129}, {3., 13.3012}}	{39.9174, 32.8096}	inf	5519.	3725.96	{2189.96, 4403.44}	86.9903	0.944735	newly	13230.	3492.87	{1243., 3299.}	83.02	0.797371	Reff	0.264038	0.512395	{0.109431, 0.8713}	133.272	ar	97541.	184581.	{174435., 193803.}	89.2342
16	R0	{1.42094, 10.5871}	{2.74772, 3.83351}	{{2.24375, 0.281379}, {3., 6.03051}}	{93.3731, 63.7908}	inf	7009.	5260.31	{2604.71, 10062.9}	83.7607	0.842028	newly	12953.	9295.93	{1974., 19140.}	90.175	0.569742	Reff	0.417131	0.636085	{0.211516, 1.16114}	84.2446	ar	93044.	187224.	{168403., 223283.}	101.221
17	R0	{1.45515, 6.16466}	{2.51421, 5.20027}	{{1.61516, 3.92151}, {3., 6.408}}	{72.7801, 19.1281}	inf	6217.	2637.63	{135.773, 2837.3}	89.0135	1.26978	newly	20574.	3201.25	{107., 5119.}	91.4011	1.31346	Reff	0.356002	0.386906	{0.0178166, 0.58356}	58.9859	ar	93254.	171601.	{157871., 203300.}	84.5646
18	R0	{2.85669, 13.2416}	{2.00955, 5.80272}	{{0.719866, 2.96491}, {3., 8.48072}}	{34.9721, 56.1781}	inf	3137.	2384.77	{202.371, 2334.04}	94.8488	1.58742	newly	17189.	1972.21	{127., 1520.}	92.3085	1.32042	Reff	0.132179	0.310924	{0.0401698, 0.484625}	165.522	ar	99644.	180402.	{170843., 189036.}	81.0462
19	R0	{2.56417, 17.9558}	{2.77504, 2.80294}	{{2.25459, 0.0619807}, {3., 4.7755}}	{14.0143, 84.3898}	inf	6398.	3439.59	{262.977, 4984.96}	92.1186	1.37699	newly	14542.	4962.3	{399., 5035.}	86.137	0.840851	Reff	0.282084	0.532397	{0.208721, 0.99428}	100.049	ar	99389.	172341.	{159373., 184484.}	73.4002
20	R0	{2.27517, 11.1864}	{1.9548, 6.04376}	{{0.740185, 3.24816}, {3., 8.35111}}	{43.7804, 45.9723}	inf	3799.	2578.64	{276.529, 2309.19}	94.2924	1.50582	newly	16864.	2185.42	{162., 1966.}	91.982	1.31132	Reff	0.172117	0.374557	{0.0676935, 0.53173}	151.557	ar	98894.	172486.	{161477., 187251.}	75.1341
21	R0	{2.35735, 10.4735}	{2.40098, 5.22908}	{{1.60044, 3.83866}, {3., 6.36216}}	{27.3785, 50.0732}	inf	3960.	1875.32	{138.494, 2428.19}	94.3821	1.51393	newly	18543.	2524.41	{130., 1773.}	94.0073	1.3171	Reff	0.172039	0.335249	{0.0285879, 0.480407}	127.618	ar	99036.	183711.	{171217., 202290.}	85.4991
22	R0	{1.79551, 11.9179}	{2.59279, 5.93417}	{{1.51403, 3.6901}, {3., 10.8687}}	{50.3766, 56.9531}	inf	5574.	10283.2	{1994.27, 15822.8}	77.8802	0.711977	newly	14003.	11199.9	{1602., 37800.}	73.9542	0.495005	Reff	0.270045	0.661633	{0.0876457, 0.918674}	167.115	ar	97033.	194631.	{171883., 263853.}	100.583
23	R0	{1.23545, 6.31687}	{2.50913, 9.35797}	{{1.93064, 4.22415}, {3., 14.3491}}	{103.094, 63.7748}	inf	4400.	6211.03	{1617.53, 11066.7}	66.2163	0.551936	newly	13260.	6479.48	{1429., 10949.}	86.0198	0.668883	Reff	0.306775	0.893327	{0.487827, 1.93062}	191.2	ar	88739.	209162.	{183345., 294196.}	135.705
24	R0	{2.24089, 10.1789}	{2.11303, 5.89189}	{{0.726506, 2.9486}, {3., 8.40081}}	{42.59, 42.1167}	inf	5491.	2136.63	{456.166, 1291.36}	96.1638	1.54583	newly	20012.	2030.22	{464., 1149.}	96.5288	1.39355	Reff	0.242442	0.354209	{0.0836268, 0.502668}	84.7748	ar	98868.	175408.	{167318., 187116.}	77.4159
25	R0	{1.57298, 10.2607}	{2.18017, 5.72878}	{{0.322623, 1.48805}, {3., 18.1022}}	{84.4504, 77.7738}	inf	7361.	9534.13	{1109.1, 32128.}	95.4421	0.921712	newly	15457.	9518.18	{839., 31808.}	100.041	0.770369	Reff	0.403627	0.438467	{0.0756824, 0.857212}	75.4826	ar	94957.	194141.	{170064., 248879.}	104.452
26	R0	{1.2154, 8.2576}	{2.5395, 6.22698}	{{1.72519, 3.24743}, {3., 8.2944}}	{108.944, 24.8522}	inf	5404.	4046.46	{1059.85, 3781.58}	90.1804	0.908593	newly	11730.	5254.55	{661., 4469.}	101.409	0.897847	Reff	0.35796	0.451983	{0.105465, 0.784955}	56.7129	ar	88421.	172112.	{159322., 191963.}	94.6502
27	R0	{2.22841, 18.7983}	{2.60451, 5.6387}	{{1.56946, 2.99478}, {3., 8.75927}}	{30.2125, 70.0042}	inf	5503.	5441.78	{741.699, 6123.42}	90.9922	1.18065	newly	11908.	6377.87	{540., 6908.}	103.972	0.845741	Reff	0.242317	0.390377	{0.100048, 0.730822}	84.2382	ar	98756.	174337.	{160979., 186398.}	76.5329
28	R0	{1.27778, 5.36767}	{2.35517, 5.077}	{{1.61111, 3.8717}, {3., 6.33306}}	{84.3174, 22.8925}	inf	5294.	1866.86	{536.709, 2320.4}	90.3478	1.11091	newly	18063.	2103.84	{390., 2261.}	92.8663	1.20442	Reff	0.340107	0.30482	{0.013953, 0.458513}	58.7094	ar	89843.	181308.	{169561., 221845.}	103.535
29	R0	{2.38042, 15.8901}	{2.6717, 9.49939}	{{1.94654, 3.94584}, {3., 14.9642}}	{22.4436, 40.2182}	inf	6676.	5203.23	{1268.23, 8289.3}	86.3848	1.06764	newly	15388.	5749.9	{929., 4999.}	94.8522	0.8791	Reff	0.307407	0.771227	{0.5051, 1.07114}	150.881	ar	99112.	184300.	{166253., 259188.}	85.9514
30	R0	{2.53188, 12.7607}	{2.22782, 4.56432}	{{1.61917, 0.0408509}, {3., 6.48157}}	{31.2379, 64.2314}	inf	7079.	2483.59	{227.794, 2792.62}	94.3631	1.52683	newly	19690.	2655.79	{190., 3786.}	92.0243	1.26078	Reff	0.319143	0.293146	{0.00939694, 0.703841}	62.6841	ar	99317.	177371.	{164375., 196014.}	78.5909
31	R0	{1.24482, 10.5471}	{2.58819, 11.6611}	{{1.75691, 2.88375}, {3., 18.0513}}	{107.917, 59.296}	inf	5495.	2832.39	{145.174, 3761.53}	84.0694	1.02459	newly	9931.	1872.56	{144., 3359.}	84.5941	1.03364	Reff	0.364384	0.702317	{0.10974, 1.72106}	124.048	ar	89097.	177619.	{158967., 232689.}	99.3546
32	R0	{2.30446, 17.9607}	{2.58947, 5.23212}	{{1.54178, 3.6862}, {3., 11.0619}}	{25.5978, 70.869}	inf	6256.	9645.72	{1962.75, 12388.7}	84.1637	0.877841	newly	13001.	12256.1	{1330., 39011.}	96.1321	0.523097	Reff	0.270705	0.677676	{0.106329, 1.53951}	171.168	ar	99028.	209298.	{183064., 290924.}	111.352
33	R0	{2.31579, 11.8236}	{2.46605, 6.51741}	{{1.61601, 2.48439}, {3., 10.5418}}	{27.5613, 51.6285}	inf	3005.	10010.5	{3240.06, 25560.3}	75.1025	0.732817	newly	15166.	12720.9	{1998., 30981.}	102.56	0.626658	Reff	0.14124	0.485677	{0.0938243, 1.35395}	255.958	ar	99012.	211044.	{184657., 304653.}	117.351
34	R0	{1.6678, 10.6744}	{2.49581, 12.9101}	{{1.47296, 3.97374}, {3., 19.4184}}	{55.571, 57.1918}	inf	4965.	11892.9	{2222.63, 18052.9}	77.5743	0.601019	newly	13645.	7649.93	{1674., 23716.}	86.9678	0.637731	Reff	0.259643	0.720868	{0.109535, 1.10486}	198.295	ar	95809.	206671.	{177956., 285493.}	115.712
35	R0	{1.65476, 7.07559}	{2.41946, 5.08272}	{{1.60747, 3.81365}, {3., 6.62833}}	{48.2084, 28.1654}	inf	5229.	1845.98	{262.795, 2446.28}	92.3533	1.33284	newly	19217.	1968.79	{202., 2480.}	92.4529	1.2973	Reff	0.270404	0.318755	{0.0982505, 0.411784}	58.1628	ar	95746.	187799.	{173459., 210040.}	96.1425
36	R0	{2.71807, 18.5959}	{2.62904, 6.46927}	{{1.55018, 3.58225}, {3., 11.7578}}	{16.219, 65.2113}	inf	7257.	8998.73	{1791.19, 11864.}	89.7402	1.032	newly	15024.	8827.36	{1039., 9742.}	86.4949	0.635168	Reff	0.322961	0.754545	{0.350858, 1.08609}	141.096	ar	99540.	200346.	{177712., 264369.}	101.272
37	R0	{2.6202, 12.7552}	{2.33934, 10.7972}	{{0.159877, 3.62683}, {3., 15.6384}}	{26.0839, 37.4748}	inf	5145.	7851.25	{2088.25, 11693.5}	79.9546	0.830559	newly	18951.	7959.62	{1634., 10001.}	89.958	0.760009	Reff	0.212734	0.768023	{0.0624896, 1.12208}	279.071	ar	99426.	195595.	{175207., 252388.}	96.7238
38	R0	{1.47672, 9.81651}	{2.73486, 4.89675}	{{2.23914, 0.18849}, {3., 6.25436}}	{85.1982, 50.1172}	inf	5442.	3899.51	{2225.25, 8333.38}	83.8541	0.91949	newly	13535.	5709.53	{1629., 11536.}	81.5113	0.719335	Reff	0.299449	0.580386	{0.15095, 1.17141}	108.641	ar	93815.	168785.	{152315., 183658.}	79.9127
39	R0	{1.90004, 12.3313}	{2.49372, 6.9695}	{{1.48772, 3.61372}, {3., 10.8275}}	{42.8578, 50.7949}	inf	6237.	9447.1	{1943.65, 15681.5}	83.2796	0.791781	newly	15042.	10060.8	{1129., 15269.}	84.9724	0.600779	Reff	0.303056	0.629256	{0.137279, 0.941523}	126.639	ar	97413.	203161.	{180023., 271161.}	108.556
40	R0	{2.64218, 18.5237}	{2.48928, 9.50067}	{{1.95714, 4.22379}, {3., 14.6338}}	{19.3294, 48.7108}	inf	4328.	6263.	{1872.22, 8851.37}	85.3422	1.00009	newly	13194.	7847.5	{1089., 21713.}	98.6186	0.757858	Reff	0.17626	0.849349	{0.435939, 1.95712}	385.672	ar	99474.	202169.	{175108., 288547.}	103.238
41	R0	{1.6945, 7.17996}	{2.30166, 4.86225}	{{1.60913, 3.98396}, {3., 6.56577}}	{47.6508, 32.2803}	inf	7462.	2735.9	{851.542, 2164.26}	92.6573	1.25186	newly	23349.	3021.5	{862., 1847.}	94.034	1.23565	Reff	0.379415	0.355116	{0.0559833, 0.645563}	53.0274	ar	96332.	174633.	{164559., 230643.}	86.7701
42	R0	{1.84559, 18.0074}	{2.49578, 9.87978}	{{0.032016, 4.07078}, {3., 14.5672}}	{56.98, 45.1349}	inf	6919.	2364.98	{799.833, 2337.6}	95.0965	1.31382	newly	10541.	1649.63	{464., 2334.}	89.6064	1.06885	Reff	0.338389	0.465544	{0.0171254, 1.05685}	82.6901	ar	97358.	176205.	{167831., 186106.}	80.987
43	R0	{2.24965, 17.0361}	{2.53379, 6.15543}	{{1.52987, 3.59992}, {3., 10.8753}}	{27.9671, 63.8683}	inf	7490.	12062.5	{722.464, 49641.}	89.2897	1.06505	newly	14237.	12564.1	{470., 41249.}	113.41	0.777269	Reff	0.339337	0.725768	{0.345232, 0.969761}	122.006	ar	98770.	202377.	{170886., 314745.}	104.897
44	R0	{2.54324, 15.7967}	{2.61045, 11.7812}	{{1.53099, 4.06092}, {3., 19.5395}}	{17.3484, 43.08}	inf	3895.	8357.3	{1555.49, 11453.6}	83.3303	0.900048	newly	14170.	6422.34	{1153., 11258.}	92.6172	0.772153	Reff	0.167193	0.636684	{0.207943, 0.866557}	285.976	ar	99354.	192015.	{169904., 276176.}	93.2633
45	R0	{2.54943, 14.8032}	{2.61979, 6.69341}	{{2.25362, 2.29789}, {3., 10.0565}}	{19.1552, 54.784}	inf	4134.	6781.21	{2594.84, 7788.13}	86.1645	0.920789	newly	14996.	8849.23	{2100., 10084.}	89.8255	0.593859	Reff	0.179352	0.81443	{0.165032, 1.37928}	366.889	ar	99380.	199046.	{179052., 205940.}	100.288
46	R0	{2.37755, 12.6461}	{2.32424, 5.04322}	{{1.60513, 3.89839}, {3., 6.36999}}	{31.2572, 60.1203}	inf	5896.	2422.84	{427.436, 4121.56}	93.3176	1.45087	newly	17991.	3130.02	{367., 5415.}	88.0745	1.18508	Reff	0.261673	0.376612	{0.00337505, 0.763358}	91.0996	ar	99096.	176589.	{166401., 197819.}	80.5821
47	R0	{1.69279, 11.6636}	{2.01874, 5.40969}	{{0.333698, 1.51052}, {3., 17.6255}}	{73.5328, 76.1252}	inf	6849.	11379.4	{356.083, 55049.3}	98.6077	1.16958	newly	14547.	13764.4	{264., 58616.}	133.557	1.01438	Reff	0.3499	0.425336	{0.079746, 0.669177}	80.372	ar	96214.	189452.	{159386., 314712.}	96.9067
48	R0	{1.82402, 10.0038}	{2.29262, 6.98478}	{{0.609552, 2.40653}, {3., 10.7575}}	{52.1946, 35.9641}	inf	6918.	7312.76	{4147.52, 10144.1}	74.4364	0.649512	newly	17877.	8804.9	{2933., 14241.}	72.4919	0.524053	Reff	0.343281	0.755598	{0.187972, 0.960895}	134.826	ar	97076.	204356.	{185452., 229385.}	110.511
49	R0	{1.6424, 11.1667}	{2.48201, 13.2391}	{{1.43756, 3.99848}, {3., 18.385}}	{57.5488, 49.9859}	inf	4677.	11009.1	{2187.99, 15595.5}	81.4537	0.669104	newly	12499.	6775.94	{1250., 24161.}	93.6429	0.718644	Reff	0.249793	0.683698	{0.391688, 0.964265}	173.706	ar	95546.	204284.	{178781., 290319.}	113.807
50	R0	{2.05638, 14.3048}	{2.5339, 11.0821}	{{1.93065, 4.13125}, {3., 15.7798}}	{28.2346, 32.0455}	inf	6276.	5368.78	{1967.81, 10300.7}	83.0059	0.858805	newly	14245.	4078.81	{1355., 7303.}	77.727	0.712171	Reff	0.290299	0.79303	{0.500856, 1.07191}	173.177	ar	98194.	200892.	{185584., 211267.}	104.587
